# Supplementary figures and images for: Barriers to anti-retroviral therapy adherence among adolescents aged 10 to 19 years living with HIV in sub-Saharan Africa: A mixed-methods systematic review protocol
Source: PLoS One. 2022 Sep 30;17(9):e0273435. doi: 10.1371/journal.pone.0273435 (PMC9524658; doi:10.1371/journal.pone.0273435)

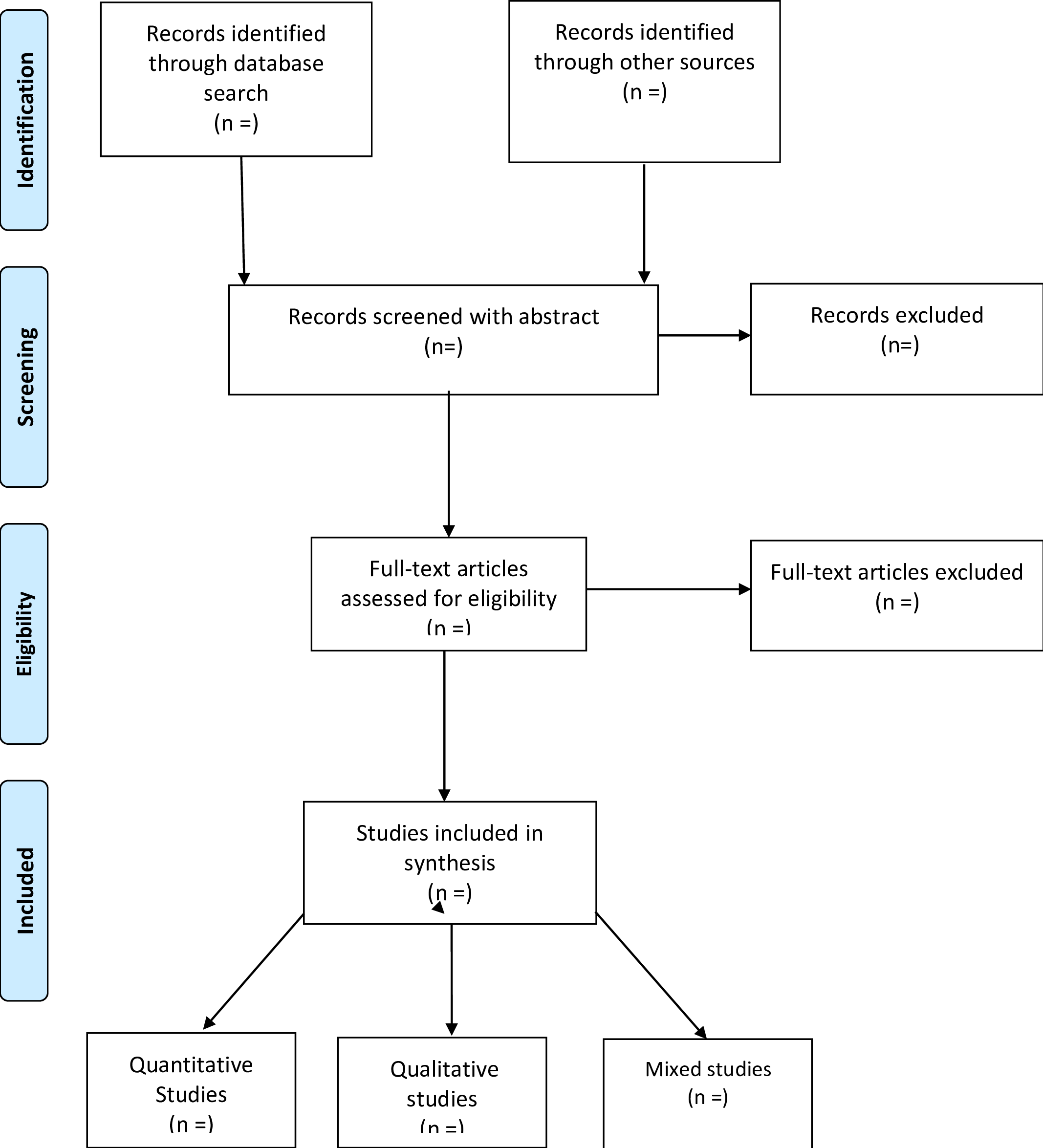

Supplement: S1 Fig — (TIF) [file pone.0273435.s001.tif]

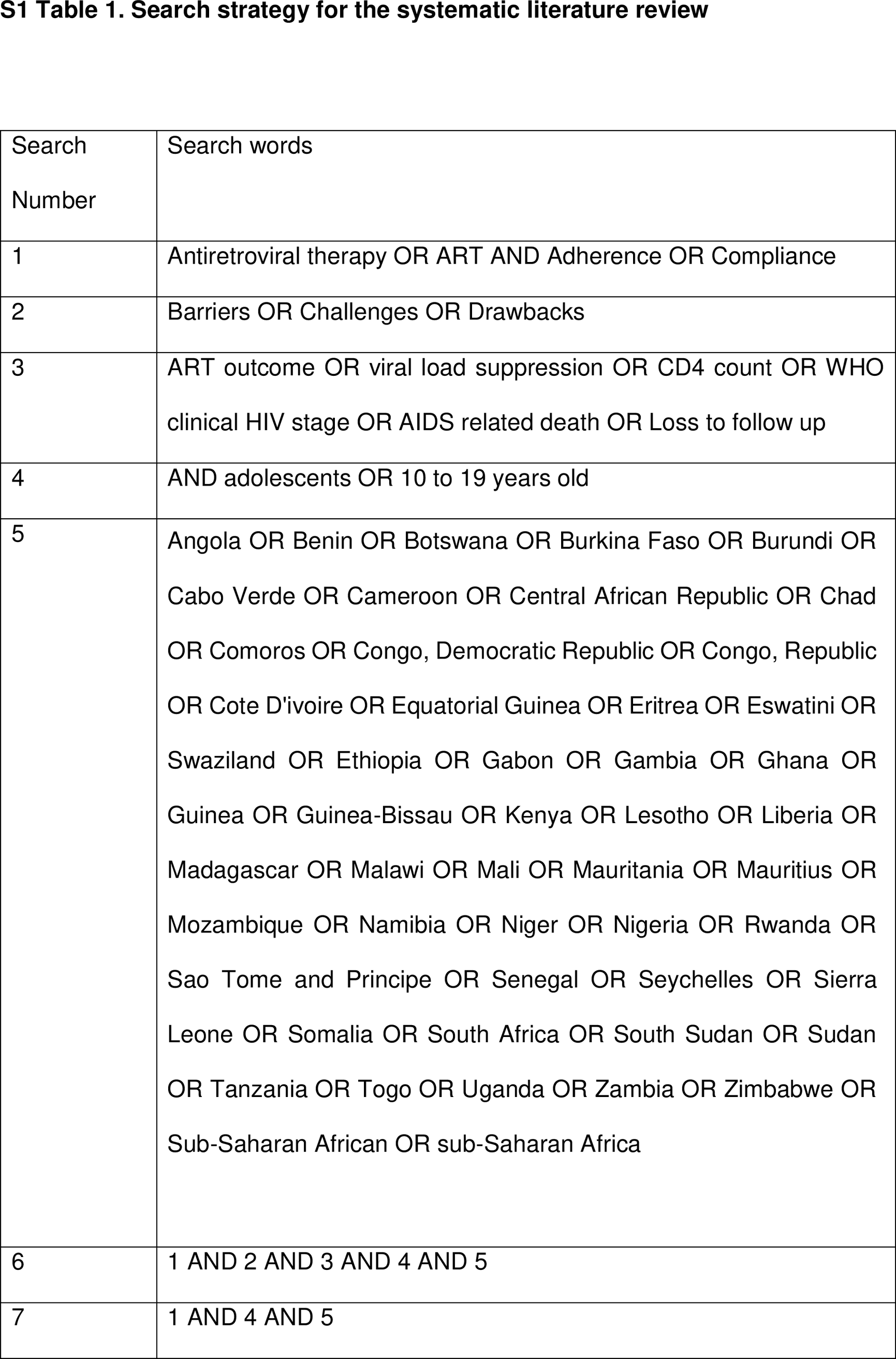

Supplement: S1 Table — (TIF) [file pone.0273435.s002.tif]

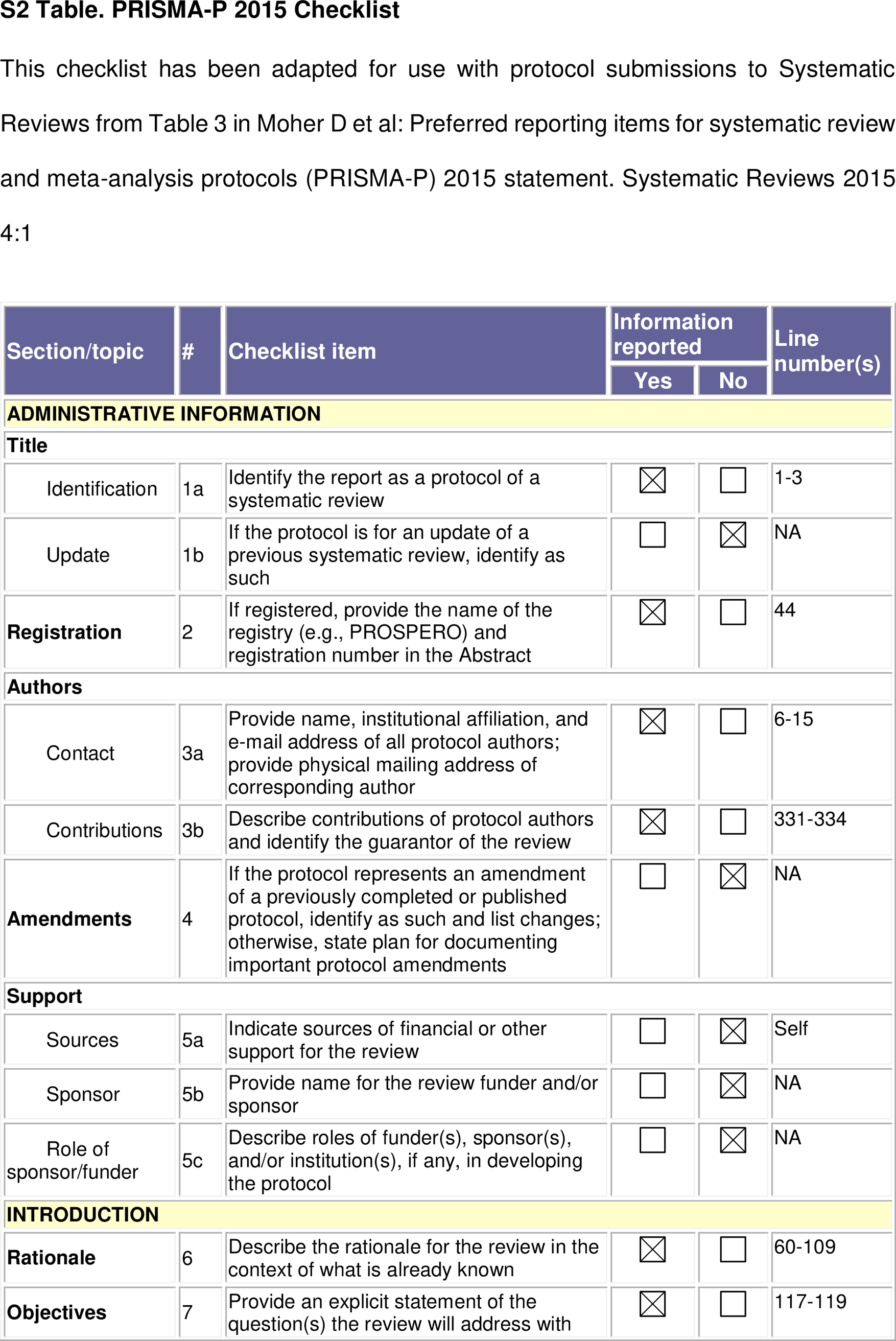

Supplement: S2 Table — (TIF) [file pone.0273435.s003.tif]
