# Supplementary material for: Barriers to anti-retroviral therapy adherence among adolescents aged 10 to 19 years living with HIV in sub-Saharan Africa: A mixed-methods systematic review protocol
Source: PLoS One. 2022 Sep 30;17(9):e0273435. doi: 10.1371/journal.pone.0273435 (PMC9524658; doi:10.1371/journal.pone.0273435)
Supplement: Attachment — Submitted filename: Response to Reviewers.docx [file pone.0273435.s004.docx]

Responses to Reviewers Comments	14^nd^ April, 2022

Dear reviewer,

We are very thankful for your valuable comments for the improvement of our manuscript entitled: “Barriers to anti-retroviral therapy adherence among adolescents aged 10 to 19 years living with HIV in sub-Saharan Africa: A mixed-methods systematic review protocol”. We have tried to address all the comments which are reflected below.

**Comment** 1: Please ensure that your manuscript meets PLOS ONE's style requirements, including those for file naming.

***Response 1****: Thanks. The authors have thoroughly edited the manuscript and are assured that it is now well aligned with the journal’s requirements. We have also edited the references into squared brackets as per the journal requirements.*

**Comment** 2**:** PLOS requires an ORCID ID for the corresponding author in Editorial Manager on papers submitted after December 6th, 2016

***Response 2:*** *Thanks.* *The ORCID ID* ***(0000-0003-2757-0663) for the*** *corresponding author has been added.*

**Comment 3**: We note that you have indicated that data from this study are available upon request.

**Response 3**: Amendment done. *The authors have revised this sentence accordingly to state that data will be available to the scientific community without restrictions for replication of study. A supporting information file of the search strategy has been attached to this manuscript (S1 Table1). On production of the main manuscript, data will be attached as s supporting information for access by anyone interested in the data.*

**Comment 4**: We note that you have referenced (ie. Bewick et al. [5]) which has currently not yet been accepted for publication.

**Response 4**: *Many thanks*. *Bewick et. al was not referenced in the study instead reference [5] is UNAIDS. Global HIV & AIDS statistics — Fact sheet , 2021, retrieved from:* [*https://www.unaids.org/en/resources/fact-sheet*](https://www.unaids.org/en/resources/fact-sheet) *(Line 70 and 361)*

**Comment 5:** Additional Editor Comments (if provided): This study is extremely important as it seeks to highlight our understanding of adherence among adolescents. The manuscript is nicely written and very easy to follow. In East and south Africa region 90% of PLHIV know their status 78% are on ART and 72% are virally suppressed. for West Africa it is 81%-77%-62%. Whilst the authors have given some data, highlighting the proportions among adolescents compared to adults makes a compelling case. In addition, the authors should correct minor typo in the methods section (pinion piece - opinion piece)

**Response 5:** *Thanks for this obeservation. The following typos have been edited including any others throught out the text. Line 5: “livng” has been corrected to “living” and in the line 172: “pinion pieces” has been corected to “opinion pieces”.*

Reviewer #1

**Comment 1:** The reviwer noted that authors have declared that data will be available on request.

***Response 1:*** *The authors have revised and stated clearly that data of this study will be available without any restrictions. A supporting information file, S1 Table2: Search strategy for the systematic literature review has been attached. When the full manuscriot is compiled, we will include data as supporting information (Line 199).*

**Comment 2:** The manuscript presents the protocol of a systematic review for barriers of adherence to ARVs among HIV positive adolescents in SSA. Adherence to ARVs has been reported to be lower in adolescents and this has also been reflected in their negative treatment outcomes. This study hence seeks to understand the barriers of adherence to ARVs among adolescents in a systematic review study. The authors have chosen to review studies from 2010 when WHO reviewed its treatment guidelines.
The manuscript is well written and structured. The authors have identified the problem and the research gap. It is also novel as it is a mixed method systematic review. The methods for the review for both quantitative and qualitative studies have been well explained in the methods section. The manuscript does not have any major weakness.

***Response 2:*** *Thank you for the positive feedback.*

**Comment 3:** However, in terms of relevance, the authors indicate that the study will be the first to track barriers of ART but it is not clear how this information will be useful in changing the negative HIV treatment outcomes in adolescents. It is not clear what new knowledge this study adds to existing studies that have reported barriers of adherence to ARVs among adolescents.

***Response 3:*** Data are *sparse to support a comprehensive understanding of challanges faced by adolescents that compromise their ability to adhere to ART. We believe that diferentiated focus on adolescents issues would help unravel potential solutions targetted towasrds adolescents reformed behvaiour that would promilgate improved adherence to treatment and improve treatment outcomes. So far, data has been aggregated with adults and not stratified to adolescents making it difficult to elucidate what adolescents focused challenges are. We believe understanding specifics about adolescents adherence bahaviour is key to improved treatment compliance and outcomes. Such knowledge would support development of specific approaches that would enhance adolescents adherence to ART and ultimately lead to an improvement in treatment outcome. (Line 109-120)*

**Comment 4*:*** I recommend that the authors could consider the use of the mixed method approach to show the added value this study will have to previous research.

***Response 4***: *Many thanks for reminding us to add this point. In page 6, line 124 and 131,* w*e include the following: “The objective of this mixed method systematic review….” And “Study design: We will include quantitative, qualitative and mixed studies in the review”.*
