# Supplementary material for: Barriers to anti-retroviral therapy adherence among adolescents aged 10 to 19 years living with HIV in sub-Saharan Africa: A mixed-methods systematic review protocol
Source: PLoS One. 2022 Sep 30;17(9):e0273435. doi: 10.1371/journal.pone.0273435 (PMC9524658; doi:10.1371/journal.pone.0273435)
Supplement: Attachment — Submitted filename: Response to Reviewers.docx [file pone.0273435.s005.docx]

**Responses to Reviewers Comments**	7^th^ July, 2022

Dear Editor,

We are very thankful for your valuable comments for the improvement of our manuscript entitled: “Barriers to antiretroviral therapy adherence among adolescents aged 10 to 19 years living with HIV in sub-Saharan Africa: A mixed-methods systematic review protocol”. We have tried to address all the comments which are reflected below.

**Comment 1**.

In your manuscript you indicate you will include publications from 2010 - 2021. Could you please

A) Outline why studies published before 2010 will not be included

***Response 1A:***

*Thanks, the rationale has been explained in line 128 to 136 as follows: “Therefore this review seeks to focus on adolescents aged 10 to 19 years as per WHO definition of adolescents and secondly studies conducted within the projections and post the 2010 treatment guidelines. The criteria for including studies conducted post the 2010 treatment guidelines is due to evidence that patients starting ART at HIV advanced stage were associated with adverse HIV outcomes [31]. In addition to this, patients who were not started on ART due to a high CD4 count had poor retention in* *care and associated with high mortality and morbidity due to AIDS progression [32, 33]. As a result, we believe that with the revised eligibility guidelines* which recommended ART initiation regardless of the clinical stage, we believe studies including adolescents on ART post the 2010 guidelines *patients will present comparable barriers to ART."*

B) Update your manuscript to indicate that you will include studies published in 2022 (to keep the submissions included up to date)

***Response 1B:*** *Thanks for this valuable input. We have updated the manuscript accordingly. The revised information is on line 40, 151, 182, 219 and 341.*

**Comment 2.** In lines 294-296 the rationale for this systematic review is given with the following text: "In our knowledge, this review will be the first study to track the barriers to ART adherence among ALHIV aged 10 to 19 years in Sub-Saharan Africa after the WHO recommendations post 2006."

However, we have identified the following systematic reviews which discuss barriers to ART adherence:

- Shubber, Z., Mills, E.J., Nachega, J.B., Vreeman, R., Freitas, M., Bock, P., Nsanzimana, S., Penazzato, M., Appolo, T., Doherty, M. and Ford, N., 2016. Patient-reported barriers to adherence to antiretroviral therapy: a systematic review and meta-analysis. PLoS medicine, 13(11), p.e1002183. [https://journals.plos.org/plosmedicine/article?id=10.1371/journal.pmed.1002183](https://eur03.safelinks.protection.outlook.com/?url=https%3A%2F%2Fjournals.plos.org%2Fplosmedicine%2Farticle%3Fid%3D10.1371%2Fjournal.pmed.1002183&data=05%7C01%7C%7C531af6e9e7ba4249851108da44a12b5c%7Ca6fa3b030a3c42588433a120dffcd348%7C0%7C0%7C637897756286765998%7CUnknown%7CTWFpbGZsb3d8eyJWIjoiMC4wLjAwMDAiLCJQIjoiV2luMzIiLCJBTiI6Ik1haWwiLCJXVCI6Mn0%3D%7C3000%7C%7C%7C&sdata=YQk3Xrhky6LWCTdHNDYRjezc6OlzoqWubQTGSjTPiYM%3D&reserved=0)
- Kim, S.H., Gerver, S.M., Fidler, S. and Ward, H., 2014. Adherence to antiretroviral therapy in adolescents living with HIV: systematic review and meta-analysis. AIDS (London, England), 28(13), p.1945 [https://journals.lww.com/aidsonline/fulltext/2014/08240/adherence_to_antiretroviral_therapy_in_adolescents.12.aspx](https://eur03.safelinks.protection.outlook.com/?url=https%3A%2F%2Fjournals.lww.com%2Faidsonline%2Ffulltext%2F2014%2F08240%2Fadherence_to_antiretroviral_therapy_in_adolescents.12.aspx&data=05%7C01%7C%7C531af6e9e7ba4249851108da44a12b5c%7Ca6fa3b030a3c42588433a120dffcd348%7C0%7C0%7C637897756286765998%7CUnknown%7CTWFpbGZsb3d8eyJWIjoiMC4wLjAwMDAiLCJQIjoiV2luMzIiLCJBTiI6Ik1haWwiLCJXVCI6Mn0%3D%7C3000%7C%7C%7C&sdata=icctIw9htfWv9UPnqFslwtDGR46LcBwnxl34IjPX2jU%3D&reserved=0)
- Ammon, N., Mason, S. and Corkery, J.M., 2018. Factors impacting antiretroviral therapy adherence among human immunodeficiency virus–positive adolescents in Sub-Saharan Africa: a systematic review. Public health, 157, pp.20-31. [https://www.sciencedirect.com/science/article/abs/pii/S0033350617304237?via%3Dihub](https://eur03.safelinks.protection.outlook.com/?url=https%3A%2F%2Fwww.sciencedirect.com%2Fscience%2Farticle%2Fabs%2Fpii%2FS0033350617304237%3Fvia%253Dihub&data=05%7C01%7C%7C531af6e9e7ba4249851108da44a12b5c%7Ca6fa3b030a3c42588433a120dffcd348%7C0%7C0%7C637897756286765998%7CUnknown%7CTWFpbGZsb3d8eyJWIjoiMC4wLjAwMDAiLCJQIjoiV2luMzIiLCJBTiI6Ik1haWwiLCJXVCI6Mn0%3D%7C3000%7C%7C%7C&sdata=2wO0fTDwSEHoU7iCo3rdOlVeEgOrcocnQa053ygRz%2FM%3D&reserved=0)

We are aware that these may differ slightly in population, and that the most recent of these reviews is from 4+ years ago, however, can you please cite these systematic reviews and outline how your work will advance on these works.

***Response 2:*** *Thanks for this valuable comment. In the tracking manuscript line 120-136, we have included a paragraph citing these studies. It reads: “So far most data has been aggregated with adults and not stratified to adolescents making it difficult to elucidate what adolescents focused challenges are [28]. Secondly, available data on adolescents even if stratified by the different age groups, included studies conducted before the 2010 WHO treatment guidelines whereby treatment elligibility was based on the WHO HIV infection stages [29]. Lastly, other studies classify adolescence as aged 12 to 24-year-old which include young people as per the WHO definition of adolescents and young prople [30] . Therefore this review seeks to focus on adolescents aged 10 to 19 years as per WHO definition of adolescents and secondly studies conducted within the projections and post the 2010 treatment guidelines. The criteria for including studies conducted post the 2010 treatment guidelines is due to evidence that patients starting ART at HIV advanced stage were associated with adverse HIV outcomes [31].In addition to this, patients who were not started on ART due to a high CD4 count had poor retention in care as well as high mortality and morbidity [32, 33]. As a result, we believe that with the revised eligibility guidelines* which recommended ART initiation regardless of the clinical stage, we believe studies including adoelscents on ART post the 2010 guidelines *patients will present comparable barriers to ART."*

**Comment 3.** Please review your reference list to ensure that it is complete and correct. If you have cited papers that have been retracted, please include the rationale for doing so in the manuscript text, or remove these references and replace them with relevant current references. Any changes to the reference list should be mentioned in the rebuttal letter that accompanies your revised manuscript. If you need to cite a retracted article, indicate the article’s retracted status in the References list and also include a citation and full reference for the retraction notice.

***Response 3:*** *This has been noted with thanks. In the tracking manuscript, line 460-464, we have revised the following reference: “”WHO 2013. Global update on HIV treatment 2013: results, impact and opportunities: WHO report in partnership with UNICEF and UNAIDS. [Internet]. WHO Press. 2013 [cited 2022 Jun 17]. Available from: https://apps.who.int/iris/bitstream/handle/10665/85326/9789241505734_eng.pdf;jsessionid=22909B1CD56BE4CD2DAF2B462D1D0BC2?sequence=1”*
